# Inflammatory pseudotumors of the paranasal sinuses

**DOI:** 10.1016/S1808-8694(15)31104-6

**Published:** 2015-10-19

**Authors:** Guilherme de Toledo Leme Constantino, Fernando Sasaki, Raquel Aguiar Tavares, Richard L. Voegels, Ossamu Butugan

**Affiliations:** 1 Otorhinolaryngologist, endonasal endoscopic surgery fellow, HC/FMUSP; 2 Medical resident, Division of Clinical Otorhinolaryngology, Sao Paulo University Clinical Hospital; 3 Physician, collaborator of the Division of Clinical Otorhinolaryngology, HC/FMUSP; 4 Associate professor, Division of Clinical Otorhinolaryngology, HC/FMUSP; 5 Associate professor, Division of Clinical Otorhinolaryngology, HC/FMUSP

**Keywords:** pseudotumor, sinuses, inflammatory, paranasal

## Abstract

Inflammatory pseudotumors may be defined as lesions that clinically and radiologically simulate neoplasms. These tumor are not a single clinical-pathological entity, but rather a generic term applied to any nonspecific, chronic, inflammatory expanding lesion. There are few reports of inflammatory pseudotumors in the nasal cavity and paranasal sinuses.

**Case report:**

We report three cases of inflammatory pseudotumors of the nose and paranasal sinuses seen at the Division of Otolaryngology of the Medical School University Hospital, Sao Paulo University.

**Discussion:**

Inflammatory pseudotumors of the paranasal sinuses present a variety of symptoms according to the site.

## INTRODUCTION

Inflammatory pseudotumors may be defined as lesions that clinically and radiologically simulate neoplasms.^1^ Various terms have been used to describe these lesions, given their histological variety,^1^^,^^2^ including: inflammatory pseudotumors, plasmocytic cell granulomas, inflammatory myofibroblastic tumors, inflammatory myofibrohistiocitary proliferation, myofibroblastomas, xantogranulomas, and tumefactive fibroinflammatory lesions. These multiple terms suggest that inflammatory pseudotumors are not a single clinical and pathological entity, but rather a generic term for any non-specific chronic inflammatory expanding lesion.^2^

Some pathologists have classified these tumors, according to their origin, into: idiopathic, regenerative/post-traumatic, developmental (embryological), functional (endocrine), iatrogenic, and infectious.^3^ Idiopathic lesions are the classical presentation of these tumors, synonymous with inflammatory pseudotumors, depending on the classification that is used. Inflammatory pseudotumors are different from neoplasms not only histologically, but also by being self-limited and having the possibility of regressing spontaneously.^3^

Various sites in the body may harbor these lesions, but the lungs, the urinary tract and the intestine are the most susceptible areas.^3^ In the head and neck, they occur mostly in the orbit and the upper aerodigestive tract.^2^

Wold and Weiland first used the term tumefactive fibroinflammatory lesion to describe a fibrosclerosing lesion of the head and neck that had histological findings similar to those encountered in Riedel's thyroiditis, in sclerosing mediastinitis and in retroperitoneal fibrosis.^4^ Macroscopically, the lesions are firm, well-defined, white, grey or brownish-yellow, and non-encapsulated.^4^^,^^5^ Microscopic findings are: dense fibrous tissue associated with chronic inflammatory cells, proliferation of fibroblasts and peripheral connective tissue that matures to yield a densely collagenized and hyalinized stroma with poorly apparent vascularization.^5^

There have been few reports of inflammatory pseudotumors in the nasal cavity and the paranasal sinuses; of these, most were in the maxillary sinus.^6^ We present three cases of nasal and paranasal sinus inflammatory pseudotumors, including the methods for diagnosis and therapy; these patients were seen at the Division of Clinical Otorhinolaryngology in the Clinical Hospital of the São Paulo University Medical School (HC/FMUSP).

## CASE REPORTS

### Case 1

**Figure 1:**
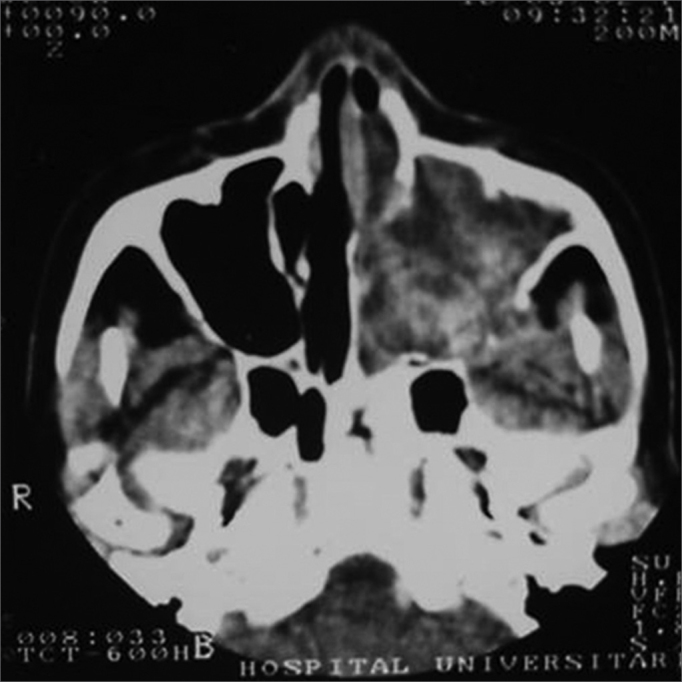
CT, axial slice, soft tissue window.

**Figure 2:**
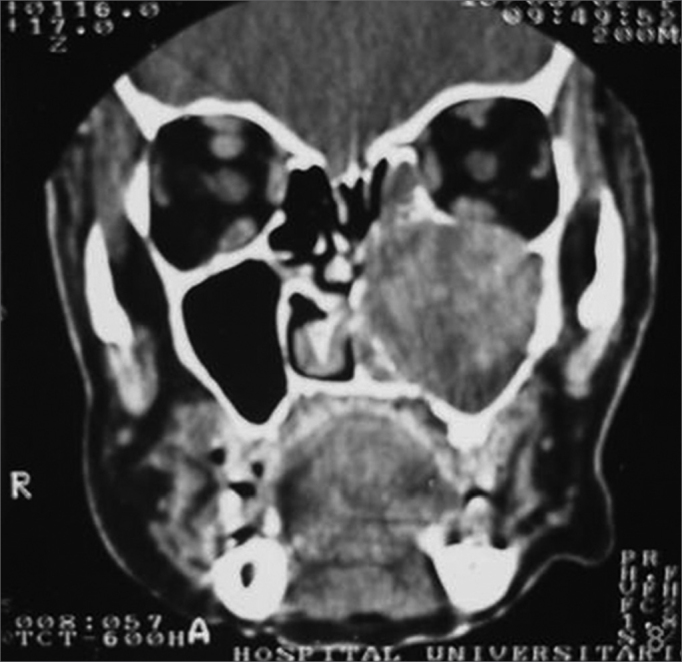
CT coronal slice, soft tissue window.

MFS, female, age 66 years, black, previously healthy, presented with purulent rhinorrhea in the left nasal fossa in the past one and a half years. The patient developed progressive nasal obstruction during the past four months in the left nasal fossa, hyposmia, pain in the left maxillary region and repeated episodes of nasal bleeding. Fever, headaches or other complaints were absent. The patient had no underlying diseases, no previous surgery, and no allergies; she used no medication, was a non-smoker and did not drink alcoholic beverages. The physical examination revealed good general health; the patient had a normal-colored mucosa and was hydrated and eupneic. Anterior rhinoscopy showed a yellowish tumor filling the left nasal fossa, and a small amount of secretion; the right nasal fossa was unaltered. Oroscopy, otoscopy and palpation of the neck were normal. The complete blood count was within normal limits. Rigid nasal endoscopy revealed a solid yellowish tumor originating from the left middle meatus. Computed tomography of the paranasal sinuses (Figure 1, Figure 2) revealed a contrast-enhanced heterogeneous lesion in the left maxillary sinus, widening of the ethmoidal infundibulum, erosion of the posterior wall and roof of the maxillary sinus, and tumor extension to the infratemporal fossa.

A tumor biopsy through the nasal fossa revealed coagulation necrosis with vascular thrombi within a polypoid structure that was partially covered with respiratory epithelium. Following this inconclusive result, a deeper sublabial biopsy was done; the procedure coursed uneventfully. This biopsy revealed necrotic tissue, and a small fragment of respiratory mucosa with fibroplasia of the chorion and a mononuclear inflammatory infiltrate; there were no signs of malignancy. Surgical treatment was undertaken, with removal of the tumor sublabially (excisional biopsy) under general anesthesia. There was erosion of the anterior wall of the left maxillary sinus and a friable, well-defined tumor that filled the sinus; the tumor was completely removed. Pathology revealed an inflammatory polyp with extensive necrosis, confirming a diagnosis of inflammatory pseudotumor.

**Figure 3:**
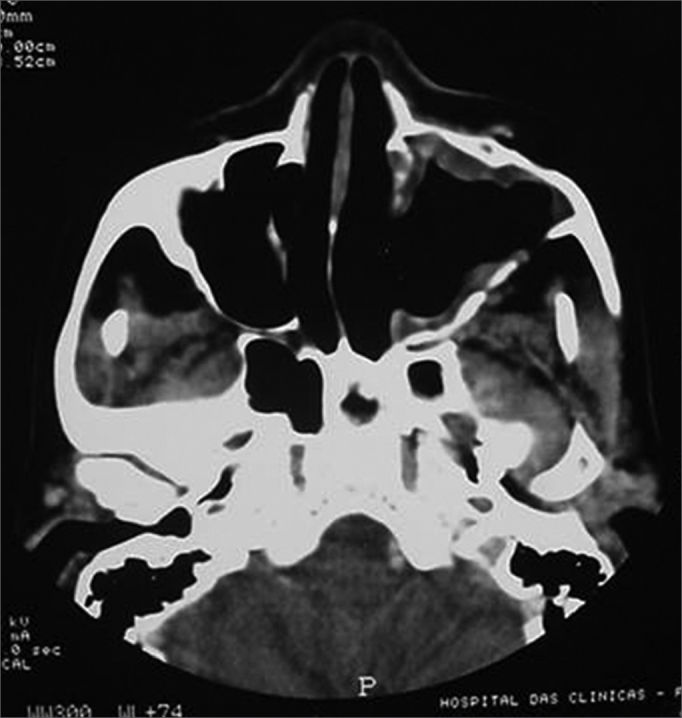
CT axial slice, soft tissue window.

**Figure 4:**
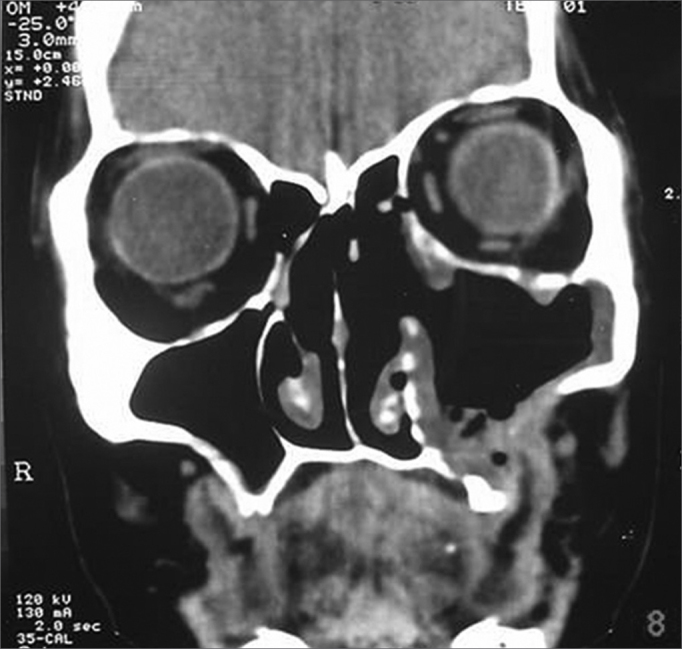
CT coronal slice, soft tissue window.

Symptoms regressed, and the patient developed a small oroantral fistula in the gingivo-labial sulcus. Telescopy revealed wide opening of the medial wall of the maxillary sinus, with no lesions in the posterior area or secretion. A control tomography six months postoperatively (Figure 3, Figure 4) showed a small lytic lesion with soft tissue components on the floor of the left maxillary sinus; this is being monitored clinically. The patient has no complaints one year and one month after surgery.

### Case 2

MIHS, female, age 62 years, white, born in the state of Bahia, and living in Sao Paulo. The patient presented bilateral, progressive nasal obstruction, worse to the right, developing over four years. She complained of frontal, pressure-like, moderately intense headaches, hyposmia, clear rhinorrhea, nasal pruritus and bleeding episodes through the right nasal fossa. The patient reported a history of systemic high blood pressure and no other disease. The patient had not been operated previously, had no allergies and did not drink alcoholic beverages. She had stopped smoking five years ago. The physical examination revealed good general health; the patient had normal colored mucosa, and was hydrated and eupneic. The arterial pressure was 130×100mmHg and the heart rate was 80bpm. Anterior rhinoscopy showed left nasal septum deviation, a hypertrophic left lower turbinate and a reddish tumor filling the entire right nasal fossa to the nasal vestibule; there was a small amount of hyaline secretion. Oroscopy, otoscopy and neck palpation were normal. Nasal telescopy brought no further information, as the tumor did not allow progression of the endoscope. The complete blood count was within normal limits; there was mild hypergammaglobulinemia (22.3% or 1.87 g/dL; reference values are 12 to 21% or 0.7 to 1.6 g/dL). Computed tomography of the paranasal sinuses revealed a large, heterogeneous expanding solid tumor in the right maxillary sinus, extending to the right nasal fossa; the tumor widened the right nasal fossa and displaced the septum towards the left. The lesion also extended to the choana, the rhinopharynx, the sphenoidal sinus and the ethmoidal cells, bulging the papyraceous lamina.

Three biopsies were done of the nasal fossa tumor. The first biopsy demonstrated inflammatory polyps. The second biopsy showed a chronic, non-specific inflammatory process of the respiratory mucosa, with fibrosis of the chorion. The third biopsy revealed a chronic eosinophilic inflammatory process in the nasal mucosa, areas of squamous metaplasia of the respiratory epithelium, and fibrosis of the chorion. A sublabial approach for excisional biopsy was done to remove the entire tumor. Pathology revealed an inflammatory mucosal polyp with non-specific chronic inflammation and ample hemorrhagic, necrotic and hyalinized areas, suggesting an inflammatory pseudotumor.

Nasal obstruction and bleeding regressed; there was hypoestesia in the right maxillary area. Telescopy showed an ample cavity with no signs of recurrence to the present (one year and three months postoperatively).

### Case 3

MSM, male, age 26 years, white, born in the state of Pernambuco, single, an IBGE survey worker. The patient presented a history of nasal bulging to the left since age 12 years, together with bilateral nasal bleeding and obstruction. At that time there was facial asymmetry and left proptosis. A first surgery was done in 1991 at another unit; pathology revealed an inflammatory pseudotumor. A new surgical procedure was done in 1993 due to recurrence of the lesion. A further biopsy was done in 1996; pathology suggested paracoccidioidomycosis. The patient was treated with amphotericin B, with no improvement. In 1998, the patient was seen at the HC/FMUSP. At this time the patient reported that nasal obstruction and bleeding had regressed, but there was still proptosis to the left. There was no diplopia or altered visual acuity.

The physical examination showed facial asymmetry and left proptosis. Nasal telescopy revealed an ample nasal cavity with signs of previous surgery and no tumors within. The complete blood count was within normal limits.

**Figure 5:**
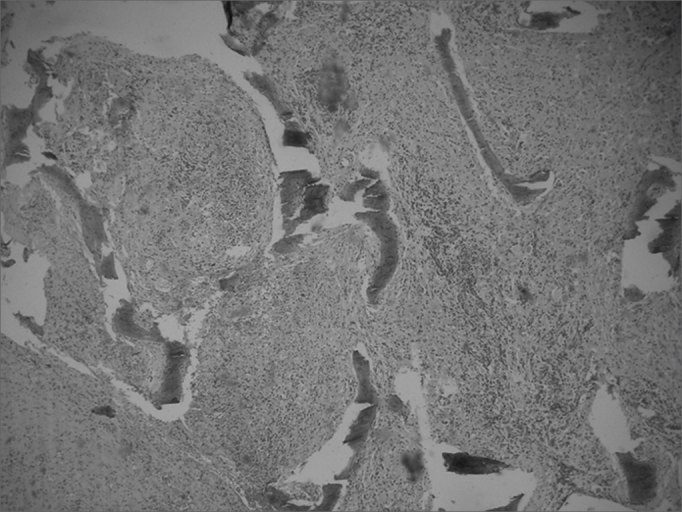
Histological sections showing a non-specific chronic inflammatory process.

**Figure 6:**
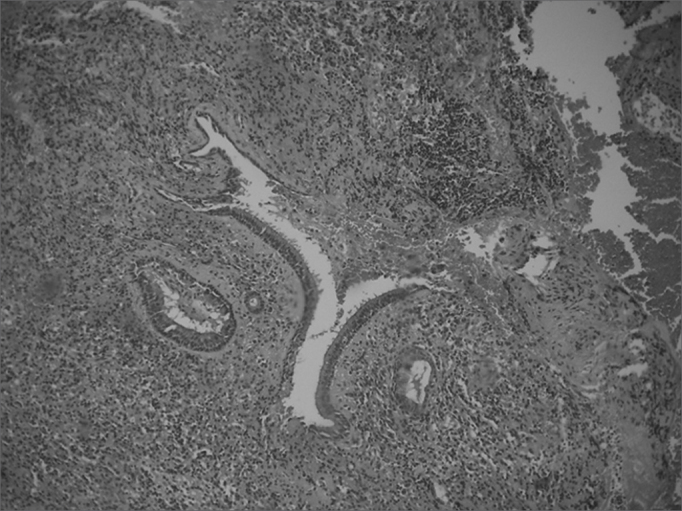
Histological sections showing a non-specific chronic inflammatory process.

Computed tomography of the paranasal sinuses revealed an expanding solid lesion in the left anterior and posterior ethmoidal sinuses, extending to the left orbital area. Four biopsies were done of the ethmoidal lesion, revealing a chronic, non-specific inflammatory process with an intense lymphoplasmocytic infiltrate in all samples. On September 1999, an excisional biopsy was done through an external approach, accessing the ethomoidal sinus from the left. Pathology demonstrated a non-specific chronic inflammatory process with no granulomas (Figure 5, Figure 6). Investigation for alcohol acid-resistant bacilli and fungi was negative.

Following this result, the diagnosis was defined as inflammatory pseudotumor of the ethmoidal sinus.

The patient is clinically stable, nasal obstruction has regressed and there have been no further episodes of nasal bleeding.

## DISCUSSION

Inflammatory pseudotumors are chronic inflammatory lesions of unknown origin. They have no common identifiable cause, although some authors have assumed that any inflammatory stimulus may originate these pseudotumors.^7^ The name is given because these lesions simulate neoplasms, both clinically and radiologically.^1^^,^^8^ There are many synonyms, such as histiocytoma, xanthogranuloma, plasmocytic cell granuloma, and inflammatory myofibroblastic proliferation, due to its wide histological presentation spectrum.^2^^,^^6^^,^^8^ Histology is non-specific,^8^ showing two typical cell types: myofibroblasts and inflammatory cells. Myofibroblasts express vimentin in 99% of cases and actin in 89 to 92% of cases on immunohistochemistry. They variably express desmin (69%) and cytokeratin (36%). The inflammatory infiltrate consists of lymphocytes, plasmocytes and granulocytes in variable proportions. These cells are found within edematous connective tissue, associated with very thin capillaries. It should be realized that this description of microscopy might vary significantly among different tumors and within the same lesion.^6^

Inflammatory pseudotumors occur in various anatomical sites; the lung is the most commonly affected organ.^2^ Inflammatory pseudotumors of the head and neck occur mainly in the orbit, and less frequently in the mouth and paranasal sinuses.^1^ Often, these areas are secondarily involved by the extension of a primary tumor of the orbit.^6^

There appears to be no sex or age preference. In a review of the literature that included 28 cases of mouth and maxillary sinus inflammatory pseudotumors, Vigneswaran found a 1.5:1 male to female ratio and a mean presentation age of 33 years (ranging from 2 to 67 years).^1^ Two of the three patients in our series were female. The age ranged from 26 to 66 years; both females were aged over 60 years.

Inflammatory pseudotumors of the paranasal sinuses present a variety of symptoms depending on the site of origin. The most frequent clinical picture is a non-specific nasosinusal tumor that grows to a stable state over months or years; it may be painful and may be associated with nasal obstruction, nose bleeding, proptosis, increased lymph nodes, dysphagia or cranial nerve dysfunction.^5^^,^^6^ Variation in symptoms, edema, erythema and fever may suggest an inflammatory etiology.^7^ Systemic symptoms are usually not encountered.^6^^,^^8^ All patients in our sample presented a typical clinical picture that included progressive nasal obstruction, repeated nasal bleeding episodes and a nasal tumor. Nasal bulging, facial asymmetry and proptosis seen in patient number 3 are also typical.

Although these tumors usually follow a benign course, such clinical behavior may not be generalized to all cases. There have been reports of local recurrence in about 37% of cases in some abdominal and mediastinal inflammatory pseudotumor series, and also cases of metastases at a distance.^2^ Vigneswaran has reported a case of an inflammatory pseudotumor in the maxillary sinus that relapsed twice within two years; this author concluded that this tumor is locally aggressive, in contrast with the more benign lesions of the orbit.^1^ Case 1 of our series presented a small postoperative lesion on the floor of the maxillary sinus, with no clinical repercussion currently (13th month postoperatively). Patient 2 was recurrence-free 15 months after surgery. Case 3 required three surgeries and a number of biopsies between 1991 and 1999 due to recurrences of the lesion; since 1999, however, this patient has remained clinically stable.

Computed tomographic findings that indicate inflammatory pseudotumors of the maxilla suggest a more aggressive appearance compared to findings in such tumors located in the orbit.^8^ Soft tissue images in maxillary sinus pseudotumors are associated with erosion, remodeling, sclerosis and bone thinning, mimicking malignant tumors.^8^ We raised the question of whether patient 3 originally had a tumor of the orbit that invaded the paranasal sinuses; this, however, appears unlikely, as orbitary forms are less aggressive radiographically.

Although there are suggestive clinical features, multiple biopsies are frequently required to establish a diagnosis of an inflammatory pseudotumor. It is an exclusion diagnosis, after eliminating the possibility of benign and malignant neoplasias, collagen diseases, vasculitis, infection and other inflammatory diseases.^8^ A cytological diagnosis of inflammatory pseudotumors is difficult.^9^

Laboratory exams may be altered, such as thrombocytosis, hypochromic microcytic anemia, hypergammaglobulinemia and an increased hemosedimentation rate, which resolve following tumor resection.2 The three patients had normal complete blood counts. Protein electrophoresis was done only in case 2, revealing mild elevation of gammaglobulins.

Corticosteroids, surgery and radiotherapy have been used in the treatment of inflammatory pseudotumors. Some authors have preferred corticosteroid therapy, while others have chosen surgery as the treatment of choice.^6^^,^^7^ High-dose corticosteroid therapy usually yields a favorable response; early lesions that include lymphoid follicles tend to be more responsive to corticosteroids, while mature lesions with a more fibrous content respond less.^7^ Our first measure is to biopsy the lesion. The material is sent for histopathology and cultured for fungi. If pathology is inconclusive, we perform an excisional biopsy for a final diagnosis. Surgery was done in all three cases; corticosteroid therapy was not used.

Radiotherapy is used in selected cases, as its success rate is low; furthermore, it may induce a malignancy in a previously benign lesion.^7^

## CONCLUSION

The aggressive aspect of paranasal sinus inflammatory pseudotumors is a diagnosticr challenge. The first concern should be to exclude neoplasia; multiple biopsies may be required. Rhinologists should raise the possibility of an inflammatory pseudotumor in the differential diagnosis of sinusal tumors, given the possibility of treating this condition medically, avoiding unnecessary ample resection.
